# Investigation of Inhalation Anthrax Case, United States

**DOI:** 10.3201/eid2002.130021

**Published:** 2014-02

**Authors:** Jayne Griffith, David Blaney, Sean Shadomy, Mark Lehman, Nicki Pesik, Samantha Tostenson, Lisa Delaney, Rebekah Tiller, Aaron DeVries, Thomas Gomez, Maureen Sullivan, Carina Blackmore, Danielle Stanek, Ruth Lynfield, the Anthrax Investigation Team

**Affiliations:** Minnesota Department of Health, St. Paul, Minnesota, USA (J. Griffith, S. Tostenson, A. DeVries, M. Sullivan, R. Lynfield);; Centers for Disease Control and Prevention, Atlanta, Georgia, USA (D. Blaney, S. Shadomy, M. Lehman, N. Pesik, L. Delaney, R. Tiller);; US Department of Agriculture, Atlanta (T. Gomez);; Florida Department of Health, Tallahassee, Florida, USA (C. Blackmore, D. Stanek)

**Keywords:** anthrax, epidemiology, zoonoses, Bacillus anthracis, bacteria, United States

## Abstract

Inhalation anthrax occurred in a man who vacationed in 4 US states where anthrax is enzootic. Despite an extensive multi-agency investigation, the specific source was not detected, and no additional related human or animal cases were found. Although rare, inhalation anthrax can occur naturally in the United States.

Anthrax is a naturally occurring disease, affecting herbivores that ingest bacterial spores when consuming contaminated vegetation or soil (*1*). *Bacillus anthracis* spores are highly resistant to weather extremes and can remain viable in soil and contaminated animal products, such as bones or hides, for many years (*2*,*3*). Heavy rains or flooding can bring spores to the surface or concentrate organic material and spores in low-lying areas. As surface water evaporates, spores may attach to growing vegetation or become concentrated in soil around roots (*3*,*4*).

Humans can become infected from exposure to infected animals or contaminated animal products (including meat, hides, and hair) or to contaminated dust associated with these products (*1*). Historically, inhalation anthrax was considered an occupational hazard for those working in wool and goat hair mills and tanneries (*5*). Recently, inhalation anthrax cases have resulted from exposure to African-style drums made of animal hides and from bioterrorist attacks (*6*,*7*). State or local health departments, the Centers for Disease Control and Prevention (CDC), and the Federal Bureau of Investigation undertake epidemiologic and criminal investigations whenever a clinical isolate is confirmed as *B. anthracis*.

## The Study

On August 5, 2011, a community hospital laboratory contacted the Minnesota Department of Health to submit for identification a *Bacillus* sp. blood isolate obtained from a patient hospitalized with pneumonia. The identification of *B. anthracis* was confirmed by nucleic acid amplification and gamma phage. CDC typed the isolate as GT59 using multiple-locus variable-number tandem repeat analysis specific for 8 loci (*8*). CDC also sequenced the strain and performed whole-genome single nucleotide polymorphism analysis. The isolate was related to strains with genotypes generally associated with imported animal products and most closely related to a strain obtained from a 1965 investigation of a case of cutaneous anthrax in a worker at a New Jersey gelatin factory, which used bone imported from India (*9*,*10*).

The patient, a 61-year-old Florida resident, had begun a 3-week trip with his wife on July 11, 2011. They drove through North Dakota, Montana, Wyoming, and South Dakota, where animal anthrax is sporadic or enzootic (Figure). While traveling, they walked in national parks, collected loose rocks, and purchased elk antlers. On July 29, they drove through herds of bison and burros, frequently stopping while animals surrounded their vehicle. The man was hospitalized on August 2, following onset of illness while he was en route to Minnesota. His condition was treated with antimicrobial drugs, according to published recommendations, supplemented with anthrax immune globulin, and he fully recovered (*11*). He and his wife were interviewed to identify potential sources of exposure. The Federal Bureau of Investigation found no concern for bioterrorism.

**Figure:**
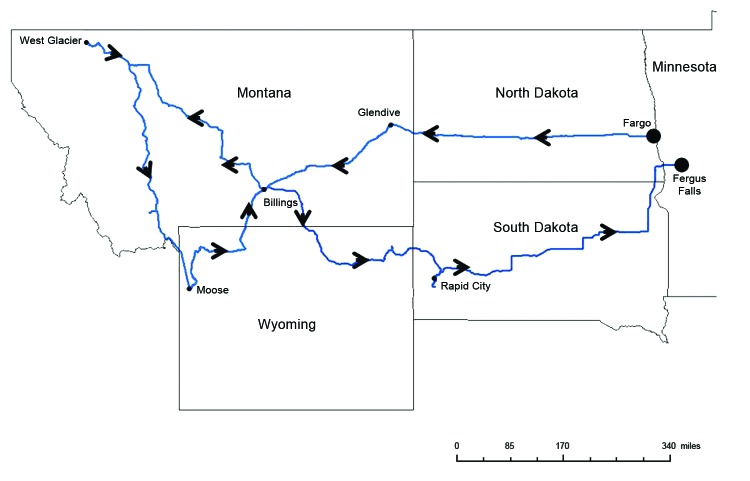
Route traveled by anthrax case-patient in the United States, July 11, 2011 to August 2, 2011.

The couple had no contact with dead animals, but reported dusty conditions while driving through the herds. The patient had not traveled abroad during the past year or been exposed to tanneries, wool or goat hair mills, bone meal, African drums, or illicit drugs. He crafted metal and stone jewelry and knives with elk-antler handles in a home workshop. One month before illness onset, he had constructed fishing flies using hair from a healthy elk he hunted in Kentucky 8 months previously.

Because his wife may have experienced the same exposure, postexposure prophylaxis was provided, including a 60-day course of oral antimicrobial drugs and a 3-dose series of Anthrax Vaccine Adsorbed (Emergent Biosolutions, Lansing, MI, USA), under an investigational new drug protocol. Symptoms of anthrax did not develop; however, she was unable to provide blood for serologic testing before starting postexposure prophylaxis.

Environmental sampling of the entire trip route was not feasible because the travel route involved several thousand miles over 3 weeks, specific suspected exposure locations were lacking, and recovering *B. anthracis* from environmental samples (*12*), particularly soil (*13*), gives variable results and is inefficient. To identify an exposure source and focus the environmental investigation, investigators obtained targeted samples from the vehicle and personal items where spores were likely to be found. From the patient’s vehicle and contents, 47 environmental samples were collected, and 18 environmental samples were obtained from his home workshop and garage (Table), following procedures of the National Institute for Occupational Safety and Health and the Environmental Protection Agency (*14*). All samples were processed and cultured by using CDC’s Laboratory Response Network culture and PCR methods at the Minnesota Department of Health and the Florida Department of Health. *B. anthracis* was not detected in any of the 65 samples.

**Table:** Samples tested for presence of *Bacillus anthracis,* 2011*

Sample type	Description (no. specimens)	PCR result	Culture result
Vacuum sock†	Floorboards (3)	ND	ND
Vacuum sock	Seats (2)	ND	ND
Macrofoam swab‡	Instrument panel (2)	ND	ND
Macrofoam swab	Arm rests (2)	ND	ND
Macrofoam swab	Dashboard (2)	ND	ND
Macrofoam swab	Steering wheel	ND	ND
	Vehicle exterior		
Macrofoam swab	Windshield	ND	ND
Macrofoam swab	Driver's window seam	ND	ND
Macrofoam swab	Hub caps (4)	ND	ND
Macrofoam swab	Intake grill	ND	ND
Bulk	Scraping of debris on wheel hubs (5)	ND	ND
Bulk	Cabin filter (2)	ND	ND
Vacuum sock	Cabin filter	ND	ND
Vacuum sock	Engine filter	ND	ND
	Personal items		
Macrofoam swab	Boots (2)	ND	ND
Bulk	Boots (2) (dirt removed from soles)	ND	ND
Macrofoam swab	Antlers (4)	ND	ND
Macrofoam swab	Fishing rod/lures (2)	ND	ND
Bulk	Rinsate of fishing lures	ND	ND
Bulk	Rinsate of rocks (6)	ND	ND
Macrofoam swab	Rock sifter	ND	ND
	Residence		
Macrofoam swab	Garage electrical box		ND
Macrofoam swab	Garage workbench surface		ND
Macrofoam swab	Electrical outlets (5)		ND
Macrofoam swab	Home air conditioner unit		ND
Bulk	Air intake filter		ND
Macrofoam swab	Rocks		ND
Macrofoam swab	Fishing gear		ND
Macrofoam swab	Mounted animal heads (2)		ND
Macrofoam swab	Computer modem		ND
Macrofoam swab	Surfaces (2)		ND
Bulk	Vacuum bag contents (2)		ND
*ND, not detected; blank spaces indicate test was not performed. †http://www.cdc.gov/niosh/topics/emres/surface-sampling-bacillus-anthracis.html. ‡http://www.ncbi.nlm.nih.gov/pmc/articles/PMC2730285/.			

Multiple agencies, including CDC, state public and animal health departments of Minnesota, North Dakota, Montana, Wyoming, South Dakota, and Florida, and other federal and state partners, conducted enhanced retrospective surveillance to identify other possible human or animal anthrax cases during June 1–August 31, 2011. Infection preventionists, medical examiners, and coroners were asked if they were aware of cases of unexplained death or fulminant illness potentially caused by anthrax. The US Department of Agriculture Veterinary Services and Wildlife Services/Animal and Plant Health Inspection Service, the Southeastern Cooperative Wildlife Disease Study, the US Geological Survey National Wildlife Health Center, the National Park Service, and state wildlife and veterinary agencies were asked to report unexplained animal die-offs and animal deaths consistent with anthrax. In addition, ≈450 Laboratory Response Network and 7 National Animal Health Laboratory Network laboratories in the participating states, the National Veterinary Services Laboratories, and other veterinary and wildlife laboratories were asked to review records for non-hemolytic *Bacillus* spp. isolates identified during the surveillance period.

No associated anthrax cases or isolates were found. A single bovine case was reported in Aurora County, South Dakota, in August 2011; however, the isolate was determined to be of an unrelated genotype (GT3). No other anthrax-associated die-offs in wildlife or domestic animals were identified, and no other suspected or confirmed human cases of anthrax were identified or reported during this period.

## Conclusions

We did not find a specific exposure associated with anthrax infection for this case-patient and no other human or related animal cases. The clinical isolate genotype and sequence closely matched several previous environmental sample isolates from North America, however, no epidemiologic links were identified. The case-patient was exposed to airborne dust while traveling through areas where anthrax was enzootic; however, testing of vehicle air filters in which the dust was concentrated was negative for *B. anthracis*. Nonetheless, the patient may have been exposed through contact with an unidentified contaminated item. This investigation was limited by the poor sensitivity expected for soil sampling and lack of data to guide additional focused environmental sampling, precluding widespread random sample collection and testing along the route traveled.

It is unusual for inhalation anthrax case-patients to have no identified exposure source (*5*,*9*,*15*). Inhalation of spore-contaminated soil has been suggested as a possible source of infection for bison in anthrax outbreaks in Canada (*16*). A heavy equipment operator acquired inhalation anthrax during a bison outbreak in Canada, where he dragged carcasses to burial sites and was exposed to airborne dust during operations (*2*).

Chronic pulmonary disease or immunosuppression may increase a person’s susceptibility to inhalation anthrax (*9*,*15*). This case-patient had a decades-long history of chemical pneumonitis, and although he reported no respiratory difficulties, perhaps his risk was increased. He also had a history of mild diabetes; diabetes has been observed in other anthrax patients (*9*).

This report highlights the challenges of investigating cases of anthrax when no specific suspected source exists. Anthrax, either naturally-occurring or bioterrorism-related, is a major public health concern, and timely recognition is critical. Clinicians and public health professionals should be cognizant that naturally acquired anthrax can occur in the United States and take appropriate steps to rapidly diagnose and investigate such cases.
